# Evaluation of Mangosteen juice blend on biomarkers of inflammation in obese subjects: a pilot, dose finding study

**DOI:** 10.1186/1475-2891-8-48

**Published:** 2009-10-20

**Authors:** Jay K Udani, Betsy B Singh, Marilyn L Barrett, Vijay J Singh

**Affiliations:** 1 Medicus Research LLC, Northridge, CA 91325, USA; 2 UCLA School of Medicine, Department of Medicine, Los Angeles, CA 90024, USA; 3 Pharmacognosy Consulting, Mill Valley, CA 94941, USA

## Abstract

**Background:**

The ability to reduce inflammation in overweight and obese individuals may be valuable in preventing the progression to metabolic syndrome with associated risks for heart disease and diabetes. The purpose of this study was to evaluate the effect of multiple dosages of a proprietary Mangosteen Juice blend on indicators of inflammation and antioxidant levels in obese patients with elevated C-reactive protein (CRP) levels.

**Methods:**

The study was an 8 week randomized, double-blind, placebo-controlled study with a pre-study 2 week washout period. The study included four groups including placebo and three difference doses of the test product, XanGo Juice™: 3, 6 or 9 oz twice daily. The primary outcome measure of this study was high-sensitivity (HS)-CRP. Secondary outcome measures included other biochemical indicators of inflammation, anthropomorphic measures and a safety evaluation.

**Results:**

One hundred twenty two (122) persons were screened for the study, 44 were randomized and 40 completed the study. HS-CRP measurements dropped after 8 weeks treatment compared to baseline in all 3 dose groups and increased in the placebo group. The changes from baseline were not significant but the comparison of change from baseline was significant for the 18 oz group when compared to placebo (p = 0.02). Other markers of inflammation (inflammatory cytokines) and a marker for lipid peroxidation (F2 isoprostane) did not show any significant differences when compared with placebo. There was a trend towards a decrease in BMI in the juice groups. There were no side effects reported in any of the groups and none of the laboratory or EKG safety assessments indicated clinically significant changes for any subject.

**Conclusion:**

In this pilot, dose-finding study, a proprietary mangosteen juice blend (XanGo Juice™) reduced CRP levels (increased change from baseline) compared to placebo for those taking the highest dose of 18 oz per day. Further studies with a larger population are required to confirm and further define the benefits of this juice. The juice was administered safely.

**Trial Registration:**

ISRCTN9300027

## Background

Obesity is a complex and difficult disorder which is multi-factorial in its etiology. Central adipose tissue is known to produce adiponectin, which plays a role in the glucose/insulin axis as well as the production of inflammatory cytokines. When central adipose tissue expands through increased fat deposition, it appears that there is a concomitant drop in the production of adiponectin along with a rise in the expression of inflammatory cytokines and C-reactive protein (CRP) [1]. Inflammation is emerging as a predictor of cardiovascular disease and may be considered a precursor of metabolic syndrome. Therefore the ability to reduce inflammation (as measured by CRP) in overweight and obese individuals may be valuable in preventing the progression to diabetes [2,3] and heart disease [4-10]. Inflammation has also been implicated in lung disease [11-13], diseases of the digestive tract [14], skin diseases [15-18], and arthritis [19-24].

There are many options in the treatment of inflammation. Steroids have been used, but are best in acute inflammatory presentation as there are potential side effects from long term use, including poor resistance to infection. Often inflammation is treated initially with nonsteroidal inflammatory drugs (NSAIDS) such as ibuprofen, naproxen and aspirin [24]. However, these drugs also have potential side effects such as gastrointestinal bleeding. A series of cyclooxygenase (COX)-2 inhibitors have been marketed in recent years, but they too have had issues related to side effects that require monitoring [24,25]. These issues were the impetus for investigation of traditional medical (Ayurveda and Traditional Chinese Medicine) herbal products which may offer products that have anti-inflammatory benefit with a lower risk-profile [26].

Mangosteen fruits, *Garcinia mangostana *L. [Guttiferae], were mentioned in Chinese medicinal records dating back to the Ming dynasty (1360 to 1644 AD). Alpha- and γ-mangostins from *G. mangostana *are identified as bioactive substances with anti-inflammatory effects [27]. The anti-inflammatory mechanism appears to be inhibition of the conversion of arachadonic acid to prostaglandin (PG)E_2 _by COX [28,29] and blocking of inhibitor kappa-B kinase (IKK) activity. IKK prevents nuclear factor kappa B (NFκB) dependent COX-2 gene transcription [30]. In addition, the γ-mangostin xanthone has been shown to inhibit lipopolysaccharide (LPS) induced activation of IKK, NFκB and human COX-2 gene promoter region dependent transcription, but had no effect on COX-1 [31]. Anti-inflammatory activity was also demonstrated in-vivo with γ-mangostin using the rat paw edema model [31].

XanGo Juice™ contains a whole fruit puree of the mangosteen in addition to other fruit juices. The purpose of this study was to evaluate the efficacy of multiple dosages of XanGo Juice compared with placebo in the improvement of inflammation and antioxidant levels in obese patients with elevated CRP levels.

## Methods

### Study Design

The study was an 8 week randomized, double-blind, placebo-controlled study with a pre-study 2 week washout period. The study was conducted at a single site: Medicus Research, LLC, in Northridge, Ca. Sample size was not calculated as this was an exploratory trial to compare three concentrations of the target product to placebo. The study was approved by the Copernicus Group IRB (Cary, NC) prior to the initiation the study.

### Recruitment

Subjects were serially recruited if they were between 30-75 years of age, had a body mass index (BMI) ≥ 30 and ≤ 45 kg/m2 (obese), a HS-CRP of ≥ 3, agreed to discontinue anti-inflammatory medications and supplements (other than daily 81 mg aspirin which was allowed), agreed to use approved birth control methods if a female of childbearing age, and agreed to not initiate or change any exercise or diet programs during the study. Subjects were excluded if they had consumed the test product in the past, had allergies to the test product, using any drugs that can affect CRP, were taking hormone replacements, anticoagulant or anti-platelet therapy, had surgery in the past 6 months, smoked cigarettes, known alcohol or drug abuse, had major systemic, inflammatory or chronic disease, untreated depression, active eating disorder, were unable to understand or follow study protocol, were pregnant or lactating and had any medical condition which in the opinion of the investigator might interfere with the subject's ability in the trial.

### Clinical Study Process

The subjects in the study came to the research clinic for a total of 4 visits (V0-V3). At (V0), the screening visit, subjects were screened for eligibility according to the inclusion/exclusion criteria. Subjects gave their informed written consent before any procedures were conducted. There was a 2 week washout phase between screening and enrollment during which subjects were asked to refrain from consuming dietary supplements (including anti-oxidants) and anti-inflammatory medications. The baseline visit (V1) took place after the washout period with subjects in a fasted state (10 hours). During this visit, the subjects underwent a physical examination, had their blood drawn and were randomized into groups. The laboratory tests and procedures included HS-CRP and a cytokine panel via Sandwich Immunoassay (Panomics, Inc., San Diego, CA), Urine F2 isoprostane (Kronos Science Laboratory, Phoenix, AZ), and safety laboratory assessments including complete blood count (CBC), comprehensive metabolic panel (CMP), and urinalysis (Primex Labs, Northridge, CA) and platelet aggregometry (Plateletworks, Helena Laboratories, Beaumont, TX). These lab tests were repeated at the 4 week (V2) and 8 week (V3) visits. An EKG was performed at baseline (V1) and again at 8 weeks (V3). During all 4 visits, anthropomorphic measures (hip and waist measurements, and weight) were obtained and vital signs (blood pressure, temperature, pulse, and respiration) were checked. Adverse event monitoring was performed at each visit using a standardized set of questions. Subjects were instructed not to change their diet nor start any new exercise program during the course of the study. In order to assess compliance, subjects were required to return all unused investigational product at each visit.

### Outcome Measures

The primary outcome measure of this study was HS-CRP, a marker of cardiac health related to inflammation. Secondary outcome measures were inflammatory cytokines, urine F2 isoprostane (a measure of lipid peroxidation) and anthropomorphic measures (weight, waist and hip measurements, BMI, and body fat percentage). Safety evaluations included CBC, CMP, EKG, urinalysis and platelet aggregation, as well as particular attention to potential gastrointestinal or cardiovascular related signals.

### Randomization

Subjects were randomized using a table of random numbers derived from a random number generating program. A simple randomization schema utilizing 4 groups was given to the clinical staff. A single master sheet was used to randomize subjects if they were eligible for inclusion in the study following the clinical screen. The master sheet included subject number, coordinator initials and date of randomization was used to track the process and insure no duplications of assignment. None of the clinical staff had access to the assignment code key.

### Product Description

The test product was XanGo Juice™ produced by XanGo, LLC. The primary ingredient of XanGo Juice was mangosteen (*Garcinia mangostana L*.) whole fruit puree. XanGo Juice also contained apple fruit juice, pear fruit juice, grape fruit juice, pear fruit puree, blueberry fruit juice, raspberry fruit juice, strawberry fruit juice, cranberry fruit juice, and cherry fruit juice. The placebo consisted of water, sucrose (3 g/30 ml), citric acid, red grape juice concentrate, fiber complex, grape skin, natural flavors, red #40, cloud (ester gum), whey protein isolate, sodium benzoate, xanthan gum, blue #1, and caramel color.

Three different dosages of the juice were tested and compared to placebo. The product doses tested were 3 oz, 6 oz and 9 oz. All doses and placebo were consumed in a total of 9 oz of liquid in identical bottles. The placebo was used to make up the volume for the lower doses. Subjects were instructed to consume the assigned drink twice a day, once in the morning and again in the evening. They therefore took a total of 0 to 18 oz of active product per day in 18 oz of fluid.

### Data Management

The data from the field activity at the Northridge, CA. site was received at the CRO facility of Medicus Research, LLC in Midlothian, VA using password protected electronic transfer. The Data Management staff logged in the data files as they were received; code books were developed to facilitate smooth and accurate editing and data entry. Statistician and Data Analyst trained the data entry personnel using the codebook as the guideline for instruction on operational processes for this data. Data was analyzed using paired sample t-tests for within subject means comparisons, independent sample t-tests for between group comparisons (Placebo vs. each of the active groups individually), t-tests of difference scores for both within and between group comparisons (Placebo vs. each of the active groups individually).

Data was analyzed with the CRO team continuing the 'blinded' structures concerning group assignment. The analysis team only had the Placebo group identified to them so that they would know how to organize the statistical comparisons. Only after the analysis was completed was the 'blind' broken in order to properly report relationships between the 3 different strengths of the product being tested and placebo.

Excel 2003 (Microsoft Corp, Redmond WA), was used for data entry, validation, restructuring, calculating changes in variables over time, reorganizing and reformatting results, and preparing graphs. Statistical analyses (descriptive statistics and means comparison tests, both within and between group, were performed using SPSS Base System ver. 16 (SPSS Inc., Chicago IL.)

## Results

One hundred twenty two persons were screened for the study, 44 were randomized and 40 completed the study. Two subjects withdrew from the study due to family related issues, and two subjects were administratively withdrawn for noncompliance. The groups were not significantly different at baseline on age, gender, CRP, BMI, or percentage body fat (Table 1). List wise deletion was utilized in the analysis of study data as each of the 4 groups had a sample size of less than 20.

**Table 1:** Baseline Demographics

	**6 oz Xango**	**12 oz Xango**	**18 oz Xango**	**Placebo**
**N**	11	12	9	8
**Male**	1	0	1	0
**Female**	10	12	8	8
**Age**	52	33	50	45
**BMI**	33.7	32.6	34.1	34.8
**Body Fat %**	41.5	39.3	37.8	39.3

Baseline characteristics of the 40 subjects who completed the study, by study group. Means are given for age, CRP levels, BMI and percentage body fat.

### Outcome Measures

#### HS-CRP

**Figure 1:**
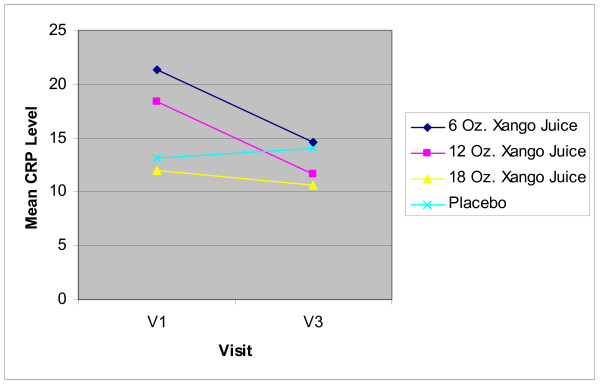
**HS-CRP**. Mean HS-CRP values at baseline (V1) and 8 weeks (V3) for all 4 groups. None of the changes from baseline nor the between group comparisons at each time point were significantly different. However the comparison of change from baseline to 8 weeks between the 18 oz group and the placebo group was significant (p = 0.019).

Mean HS-CRP measurements dropped after 8 weeks treatment compared to baseline in all 3 juice product dose groups (Figure 1). In contrast, HS-CRP measurements increased in the placebo group. None of the changes from baseline nor the between group comparisons at each time point were significantly different (Table 2). However the comparison of change from baseline to 8 weeks between the 18 oz group and the placebo group was significant (p = 0.019).

**Table 2:** HS-CRP

**Group**	**Baseline**	**8 weeks**	**Change**
**Placebo**	13.10 ± 5.2	14.00 ± 8.5	+ 0.90 ± 9.5
**6 oz Xango**	21.30 ± 16.8	14.65 ± 8.8	- 6.65 ± 11.7
**12 oz Xango**	18.33 ± 9.0	11.67 ± 4.2	- 6.66 ± 5.8
**18 oz Xango**	12.00 ± 5.6	10.67 ± 5.6	-1.33 ± 3.0

Mean HS-CRP levels plus/minus standard deviations at baseline, after 8 weeks and the change over time. None of the changes from baseline were statistically significant. However the comparison of change from baseline to 8 weeks between the 18 oz group and the placebo group was significant (p = 0.019).

#### Inflammatory Cytokines

##### Epithelial Cell-Derived Neutrophil-Activating Protein (ENA)-78

There were no significant between group differences in ENA-78 for the 6 and 18 oz groups. There was a significant differences between placebo and the 12 oz group at baseline (p = 0.002) and this difference continued for visits at 4 (p = 0.002) and 8 weeks (p = 0.022).

##### Human Interferon-Inducible Protein 10 (IP-10)

A comparison of the 6 oz juice to placebo for IP-10 found a difference only at 8 weeks, with lower levels in the placebo group (p = 0.001). There was no significant comparative change from baseline for these two groups. A comparison of the 12 oz juice to placebo indicated no significant differences between groups except for the change from baseline to the 8 week visit (p = 0.029). The comparison between the 18 oz group to placebo resulted in a significant difference between the groups at the 8 weeks visit, with lower levels in the placebo group (p = 0.016).

##### Interleukin (IL)-12p70

Between group comparisons IL-12p70 resulted in significant differences for all three juice doses in comparison to placebo at 8 weeks. Levels of IL-12p70 were comparatively decreased in all juice groups; 6 oz (p = 0.0420, 12 oz (p = 0.0120 and 18 oz (p = 0.006)

##### Platelet Derived Growth Factor (PDGF)-BB

There were no significant differences for PDGF-BB compared to placebo groups at any measurement point for the 6 oz, 12 oz, or 18 oz groups. There were also no significant comparative changes between baseline and 8 weeks.

##### CCL-5

There were no significant differences between groups for CCL5 (also known as RANTES: *Regulated upon Activation, Normal T-Cell Expressed and Secreted*) at any measurement points for the juice groups in comparison to placebo. There was a significant comparative change from baseline to 8 weeks for the 12 oz group (p = 0.027). In that case there was a comparative reduction in the 12 oz juice group.

##### Macrophage Inflammatory Protein-1 Beta (MIP-1 beta)

There were no significant differences compared to placebo for MIP-1 beta at any measurement point for the 6 oz or 12 oz groups. The 18 oz group showed a difference between baseline and 8 week visit, with a reduced measurement for the placebo group (p = 0.040).

#### Lipid Peroxidation

Lipid peroxidation was measured via F2 isoprostane levels in the urine, normalized for urine creatinine. When normalized, there were no significant changes from baseline, nor any significant differences between the 3 different dose groups compared with the placebo group at 8 weeks.

#### BMI and Body Fat Measurements

**Figure 2:**
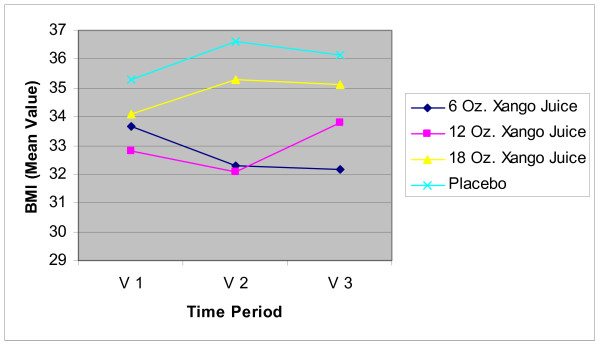
**BMI**. Mean BMI values at baseline (V1), 4 weeks (V2) and 8 weeks (V3) for all 4 groups. There were no significant differences between groups at baseline. There were significant decreases compared to placebo for subjects in the 6 oz juice group at the 8 week visit (p = 0.006), for subjects in the 12 oz group at 4 weeks and 8 weeks (p = 0.005 for both comparisons). There were no significant differences between the 18 oz group and placebo at any time point.

**Figure 3:**
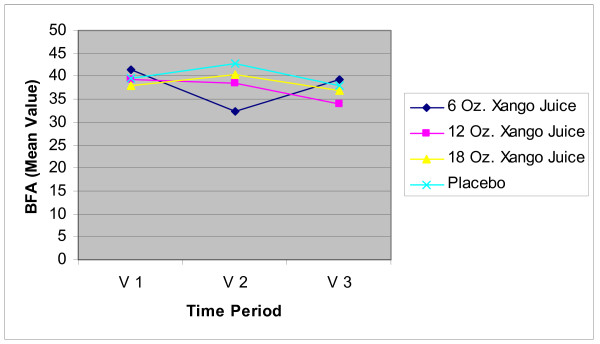
**Body Fat Analysis**. Mean values of body fat percentages at baseline (V1), 4 weeks (V2) and 8 weeks (V3) for all 4 groups. The only significant difference between groups was between the 6 oz Juice group and placebo at 8 weeks with the active product mean lower than that for placebo group (p = 0.016).

The results for BMI are presented in Figure 2 and those for body fat analysis in Figure 3. For subjects in the 6 oz juice group, there were no significant differences in BMI compared to placebo at baseline or at the 4 week visit. However, at the 8 week visit, there was a significant decrease in the juice group (p = 0.006). For subjects in the 12 oz group there was no significant difference in BMI from placebo at baseline and there was a significant decrease in compared to the placebo group at 4 weeks and 8 weeks (p = 0.005 for both comparisons). There was no difference in BMI between the 18 oz group and placebo at any measurement point.

Body fat percentages (BF) for subjects in the 6 oz juice group were not significantly different when compared to placebo at baseline or at 4 weeks. At 8 weeks, there was a significant difference in mean BF with the active product mean higher than the placebo group (p = 0.016). BF for those in the 12 and 18 oz product groups were not significantly different from placebo at baseline, 4 weeks, or 8 week measurement periods.

#### Safety Analysis

There were no side effects reported in any of the 3 treatment groups or in the placebo group. None of the laboratory or EKG safety assessments indicated clinically significant changes for any subject.

## Discussion

XanGo Juice demonstrated an ability to reduce inflammation (as measured by HS-CRP), at all 3 dosages while the placebo group showed a small increase in the amount of inflammation. When compared with placebo, the change in HS-CRP over 8 weeks was significant in the 18 oz XanGo Juice group, but not for the 6 oz and 12 oz groups. This suggests a possible dose dependent effect, but must be interpreted cautiously as the measurement variability was greater in the lower dose groups.

Other markers of inflammation (inflammatory cytokines) and a marker of lipid peroxidation (a potential indicator of oxidative stress) did not show any clinically significant differences for the juice groups when compared with placebo. There was an indication that XanGo Juice may assist in weight loss as measured with the BMI and further testing is needed to confirm this suggestion.

This study was limited by its small sample size. There were 40 subjects in the study and 4 groups, thus there were approximately 10 subjects per group. The small sample size decreases the likelihood that changes from baseline will be significant and reduces the likelihood that between group differences will be statistically significant. In addition, the use of multiple comparisons in the analysis of the study data increases the risk of statistical error resulting in a false positive. The use of an ANOVA or ANCOVA analysis, which would have been more appropriate to compare multiple groups, was precluded by the small sample size and lack of a reliable covariant. Therefore, the results of this pilot study should be interpreted as trends to be confirmed with further investigations using a larger sample size.

The XanGo Juice™ product was safe at all dosages tested. There were no adverse events (clinical, laboratory, or vital sign) attributed to the product during the course of the study.

## Conclusion

In this pilot, dose-finding study, a proprietary mangosteen juice blend (XanGo Juice) reduced CRP levels (increased change from baseline) compared to placebo for those taking the highest dose of 18 oz per day. Further studies with a larger population are required to confirm and further define the benefits of this juice. Longer studies will be needed to explore the role of inflammation in the reduction of the risk of cardiovascular disease and diabetes.

## Competing interests

XanGo LLC sponsored the study. Besides sponsorship of this study, none of the employees of Medicus Research, LLC have any other financial relationships with the company. MLB was a consultant to Medicus Research and has no direct relationship with XanGo.

## Authors' contributions

JKU and BBS were involved with the design and execution of the study. BBS and VJS managed the data and conducted the analysis. All authors participated in the writing of the manuscript.
